# The liver reconditioning in critical care medicine

**DOI:** 10.1097/ACO.0000000000001544

**Published:** 2025-06-24

**Authors:** Luigi La Via, Christian Zanza, Manfredi Tesauro, Francesca Rubulotta, Raymond Planinsic, Yaroslava Longhitano

**Affiliations:** a Department of Anaesthesia and Intensive Care, University Hospital Policlinico “G. Rodolico-San Marco”, Catania; b Department of Systems Medicine, Geriatric Medicine Residency Program – University of Rome “Tor Vergata”, Rome; c Department of Anesthesiology, Critical Care, Pain and Emergency, CHIRMED, University of Catania, Sicily, Italy; d International Women in Intensive and Critical Care Medicine Network, iWIN ETS Foundation; e Department of Anesthesiology and Perioperative Medicine, University of Pittsburgh, Pittsburgh, Pennsylvania, USA

**Keywords:** hypothermic perfusion, liver transplantation, machine perfusion, normothermic perfusion, organ preservation, organ reconditioning, viability assessment

## Abstract

**Purpose of review:**

Machine perfusion has emerged as a transformative alternative to static cold storage in liver transplantation, necessitating a comprehensive review of current evidence. This article examines recent advances in preservation techniques, therapeutic applications, and future directions of machine perfusion technologies.

**Recent findings:**

Clinical trials demonstrate superior outcomes with machine perfusion compared with conventional preservation, particularly for marginal and donation after circulatory death grafts. Different protocols – hypothermic, subnormothermic, and normothermic perfusion – show specific advantages in various clinical settings. Technology enables therapeutic interventions like defatting steatotic livers and administering cell-based therapies. Advanced monitoring systems allow real-time graft function assessment, supporting evidence-based acceptance decisions. Novel developments include artificial intelligence applications, new perfusion solutions, and blockchain technology for standardization.

**Summary:**

Machine perfusion represents a significant advancement in liver transplantation, though implementation challenges remain regarding infrastructure, training, and costs. The technology’s ability to optimize marginal grafts and enable therapeutic interventions may expand the donor pool. Future research should focus on standardizing protocols, developing cost-effective solutions, and validating emerging technologies for widespread clinical adoption.

**KEY POINTS**

- Machine perfusion provides superior outcomes compared with static cold storage, particularly for marginal and donation after circulatory death grafts, with demonstrated reductions in posttransplant complications and improved graft survival.
- Different temperature paradigms (hypothermic, subnormothermic, and normothermic perfusion) offer unique advantages, with emerging evidence supporting combination protocols for optimizing organ preservation and assessment.
- Real-time viability assessment during machine perfusion enables evidence-based decision-making through monitoring of perfusion parameters, biochemical markers, bile production, and novel biomarkers.
- Machine perfusion serves as a platform for therapeutic interventions including defatting protocols, cell-based therapies, and pharmacological treatments offering potential for organ rehabilitation before transplantation.

## INTRODUCTION

Liver transplantation is a cornerstone therapy for end-stage liver disease and select cases of hepatocellular carcinoma [1]. Even after decades of technological progress, surgical methods, and perioperative care the subject of organ shortage and quality remains a challenge in the field [2]. The discrepancy between organ demand and availability is contributing to the rising mortality of patients on the waitlist, with around 15% of patients succumbing currently before receiving a transplant [3]. To help combat this critical shortage, transplant centers have increasingly broadened their donor acceptance criteria, which mirrors the use of extended criteria donors (ECDs) and donation after circulatory death (DCD) grafts [4]. These organs, however, are more vulnerable to ischemia-reperfusion injury and have an increased risk for posttransplant complications including primary nonfunction and early allograft dysfunction (EAD) [5,6]. Static cold storage (SCS), the standard method of preservation used in clinical practice, is cheap and easy to execute but does have serious limitations in marginal graft preservation [7]. These limitations have led to the emergence of organ reconditioning strategies, which signifies a fundamental change in liver storage and optimization [8]. Reconditioning technologies, especially machine perfusion, provide the only potential opportunity to monitor and improve organ quality before transplantation [9]. Organ reconditioning encompasses many strategies, including not just hypothermic, but also normothermic machine perfusion (NMP), with its own set of benefits [10]. Mimicking these technologies not only creates a platform for organ preservation but also presents treatment options via the delivery of drugs, oxygen, nutrients, and cellular components [11]. Recent clinical trials have found favorable results from these experimental studies, suggesting better outcomes with reconditioned grafts than those preserved using conventional methods [12]. Furthermore, organ reconditioning allows for innovative approaches to transplantation medicine as discarded organs can be used, which leads to growing the donor pool [13^■^]. Assessing organ viability while it is being perfused with vasorum will help transplant teams make informed decisions, thus potentially preventing complications that may arise after the organ has been transplanted [14^■^]. Here, we provide a narrative review discussing the current status of organ reconditioning in liver transplantation along with enabling technologies, clinical applications, and future perspectives. Transplant professionals must realize these advances and continue to widen the multiple organ base, thus optimizing organ utilization and ensuring beneficial outcomes for patients undergoing transplantation [15^■^].

## METHODS

We conducted a comprehensive search and summarized the most recent clinical evidence up until 31 December 2024, on PubMed, EMBASE, and the Cochrane Library. We used MeSH terms and synonyms related to the following keywords: liver transplantation, machine perfusion, organ preservation, normothermic perfusion, hypothermic perfusion, organ reconditioning, and viability assessment, applying an English language restriction. As a result, we included 108 relevant papers for this clinical review, summarizing the latest findings.

## LIVER PRESERVATION METHODS: FROM STATIC COLD STORAGE TO DYNAMIC PRESERVATION

The evolution of liver preservation techniques represents a fundamental advancement in transplantation medicine. SCS, developed in the late 1960s, has long served as the gold standard for organ preservation [16^■^]. This technique, which is based on the principle of metabolic reduction through the use of hypothermia and specialized preservation solutions, has led to the widespread application of liver transplantation programs in many centers around the world [17^■^]. In the late 1980s a groundbreaking solution that improved upon older methods by offering superior onboard preservation capabilities [18^■^]; however, although successful, SCS has inherent limitations, especially as applied to ECDs and long periods of cold ischemia [19^■^]. There are significant concerns about lack of oxygen delivery, waste products, and inability to monitor organ viability while preserved [20^■^]. This has led to the realization that these are not without limitation, prompting the discovery of dynamic preservation methods. Moving from static to dynamic preservation is a natural progression of this methodology because it is more physiologically relevant and facilitates continuous monitoring of organ function [21]. General temperature protocols divide dynamic preservation techniques into hypothermic (0–12 °C), subnormothermic (20–25 °C), and normothermic (37 °C) machine perfusion [22]. Each temperature paradigm has its unique benefits and addresses different components of organ preservation. The method of hypothermic machine perfusion (HMP) reduces metabolism, preserves minimal but important cellular function, and washes out the inflammatory mediators [23]. Subnormothermic perfusion could be an intermediate strategy, providing some of the advantages of both hypothermic and normothermic, while minimizing metabolic consumption compared with complete normothermia [24]. These systems can be implemented with dynamic preservation systems with oxygen carriers and advanced perfusion solutions [25]. This has led to longer preservation times and better organ viability, especially for marginal grafts [26]. Moreover, the advent of portable perfusion devices has improved the practicality of these technologies used in the clinical context [27]. Recent data suggest that dynamic preservation techniques may activate protective cellular mechanisms also, including upregulation of stress proteins and anti-inflammatory pathways [28]. The biological modulation observed during preservation holds potential therapeutic implications to enhance the quality of the graft [29]. This changing paradigm from SCS to dynamic preservation strategies is perhaps one of the most consequential breakthroughs in the history of transplantation medicine, unlocking the trajectory toward not only organ preservation but also organ optimization and assessment [30]. This advancement is crucial to the future of this field because its development pushes technologies and possibilities of the usage of organic matter.

## MACHINE PERFUSION TECHNOLOGIES IN LIVER TRANSPLANTATION

Machine perfusion is an evolving process rather than a single event, particularly for abdominal organs which require different perfusion strategies. These strategies, delivered by specialized machines, range from practical clinical applications to experimental approaches based on specific medical needs. These technologies can be broadly categorized into three groups according to their operating temperatures, each category representing unique advantages and applications [31].

### Hypothermic machine perfusion

HMP at 4–10 °C constitutes the most investigated dynamic preservation approach. This method retains the protective effects of hypothermia’s metabolic suppression while perfusing the organs continuously, which has been demonstrated to minimize both oxidative stress and vascular resistance [32]. In liver DCD, minimizing damage from cold ischemia is critical, and the application of HMP has been particularly successful. Clinical data have shown that HMP effectively reduces EAD and biliary complications, with roughly 40% reductions in posttransplant complications compared with SCS [33]. Results from the landmark HOPE trial showed that dual hypothermic oxygenated perfusion substantially improved 1-year graft survival (69 vs. 95%) and reduced the incidence of posttransplant cholangiopathy as compared with conventional preservation techniques [34^■■^]. The success of HMP can be explained by microvascular circulation maintenance, inflammatory mediators washout, and metabolic substrates supply with cellular metabolism being an incidental phenomenon. Other recent studies have emphasized the crucial role of oxygen supplementation during HMP, demonstrating that perfusion with oxygen can enhance mitochondrial activity and attenuate reperfusion injury [33]. The simplicity and economic nature of the technique compared with the normothermic perfusion and method has also led to broad adoption of it in transplant centers globally [34^■■^].

### Normothermic machine perfusion

In NMP, the liver is perfused at near-physiological temperature (37 °C), with near-physiological rates of metabolism, enabling real-time assessments of the graft function [8]. The pivotal COPE trial randomized 220 liver transplants at multiple centers, showing NMP significantly decreased peak aspartate transaminase (AST) (median 49% less) as well as improved 1-year graft survival (86 vs. 69%) compared with conventional cold storage [35]. This technique has been especially useful to evaluate borderline grafts, which can be critically appraised by measuring functional endpoints such as bile output, lactate clearance, and insulin sensitivity [36]. These physiological conditions allow NMP to give clinicians real-time insights into organ function and organ viability before transplant. The simultaneous assessment of metabolic parameters has transformed the evaluation of marginal organs and allowed the use of grafts that would previously have been discarded using classical preservation standards. NMP provides a constant supply of oxygen and metabolites that are necessary for cellular repair mechanisms and endothelial integrity over the extended horizon of transplantation, which can lead to improved posttransplant outcomes.

### Subnormothermic machine perfusion

Subnormothermic machine perfusion (SMP) means operating at intermediate temperatures (20–25 °C), thus acting as a compromise between HMP and NMP. This keeps enough metabolic activity to provide viability but is less demanding from a perfusion solution perspective [37]. Clinical experience indicates that SMP provides a compromise between the metabolic suppressive benefits of hypothermia and the assessment advantages of normothermia. According to recent studies, SMP could reduce preservation injury of grafted organs and enhance the function of grafted organs, especially steatosis liver [38]. It operates in an intermediate temperature range able to maintain sufficient metabolic activity for the evaluation of organ viability but limits the logistical complexity and cost of full normothermic perfusion. Despite advances in this area, organ preservation has lagged in the development of controlled rewarming as a technique and is promising, especially in marginal organs, in minimizing the impacts of preservation injury during reperfusion. The straightforward perfusion requirements and lower metabolic requirements of SMP represent an opportunity for centers wishing to introduce machine perfusion technology while minimizing operational complexity and resource use. Table 1 summarizes the key aspects and characteristics of machine perfusion technologies in liver preservation.

**Table 1:** Comparison of machine perfusion technologies in liver preservation

Characteristics	Hypothermic machine perfusion	Subnormothermic machine perfusion	Normothermic machine perfusion
Temperature (°C)	4–10	20–25	37
Metabolic state	Suppressed metabolism	Intermediate metabolic activity	Near-physiological metabolism
Perfusion solution	Simple preservation solutions	Modified preservation solutions	Complex blood-based solutions
Key advantages	Lower cost Technical simplicity Reduced vascular resistance Well-established safety profile	Balance between suppression/assessment Less complex than normothermic machine perfusion Moderate cost Reduced logistics	Real-time viability assessment Functional evaluation Organ recovery potential Bile production assessment
Main limitations	Limited viability assessment Restricted metabolism evaluation Cold injury risk	Limited evidence Undefined parameters Intermediate benefits	High cost Technical complexity Resource intensive Warm ischemia risk

Ideally, perfusion technology should be selected based on different characteristics, such as the type of donor, intended preservation time, and logistical factors [39]. On current perfusion devices, these monitoring systems are advanced and provide continuous full-fledged perfusion data, allowing for the dynamic monitoring of graft viability [40]. The capabilities of these systems have been supplemented by the development of novel oxygen carriers and perfusion solutions [41]. Combination or sequential perfusion protocols have emerged as promising strategies, potentially harnessing a complementary benefit of different temperature paradigms [42]. As an example, the initial HOPE followed by NMP has reported good outcomes in high-risk grafts [43]. Using these techniques in combination with new therapeutic strategies, including defatting protocols or immunomodulation, creates a promising frontier for transplantation medicine [44].

## VIABILITY ASSESSMENT AND PREDICTIVE MARKERS DURING MACHINE PERFUSION

The ability to assess organ viability during machine perfusion represents a significant advantage over SCS, enabling evidence-based decision-making regarding organ utilization. Various parameters and biomarkers have emerged as potential predictors of posttransplant outcomes [12].

### Perfusion parameters

Fundamentals parameters of perfusion – vascular resistance, flow rates, and pressure gradients – are indicators of organ quality. Real-time information on graft function and vasculature integrity can be obtained by dynamic monitoring of these parameters. The best transplant outcomes are associated with two key vascular measurements: maintaining stable vascular resistance (ideally at 0.25 mmHg/ml/min) and achieving adequate blood flow rates (with portal flow exceeding 500 ml/min and arterial flow above 150 ml/min after transplantation) [45]. In particular, vascular resistance over the first 2 h of perfusion has been shown to correlate well with graft function, with a decreasing resistance over this time period associated with optimal function. Assessment of oxygen consumption during perfusion has also become an important indicator of metabolic activity and graft viability [46]. Normal metabolic function will produce oxygen consumption rates of 0.7 ml O_2_/min/kg of liver tissue; declining consumption suggests cellular viability impairment. This continuous capture of influential parameters over the storage duration is a significant advantage over traditional SCS, allowing early intervention at the onset of detrimental signs.

### Biochemical markers

Lactate clearance during NMP has been established as a reliable marker of liver function, with studies demonstrating its predictive value for posttransplant outcomes [47]. Successful grafts typically demonstrate lactate clearance to levels below 2.5 mmol/L within the first 2 h of perfusion, while failure to clear lactate often indicates compromised metabolic function. Similarly, glucose metabolism and pH stability serve as important indicators of hepatic metabolic capacity [48^■■^]. Normal perfusate pH maintenance between 7.35 and 7.45 without excessive bicarbonate requirements, coupled with stable glucose levels between 4 and 10 mmol/L, generally indicates adequate hepatic function. The assessment of transaminase levels in the perfusate provides information about hepatocellular injury, though their predictive value must be interpreted in context [49]. While elevated alanine transaminase/AST levels (>3000 IU/L) may indicate significant hepatocellular damage, these markers should be evaluated alongside other parameters. The dynamic nature of these biochemical markers during perfusion provides valuable trending information, often more useful than absolute values. Their continuous monitoring enables real-time assessment of liver metabolism and potential recovery during the preservation period, offering crucial information for transplant decision-making.

### Bile production and composition

Bile production and composition during normothermic perfusion have thus become important viability markers. As such, the volume and quality of bile output positively correlate with biliary complications after transplant [50]. Functional livers secrete bile at rates of greater than or equal to 1 ml/h during normothermic perfusion, and increasing production over time indicates improved graft function. The composition of bile, including both its pH level and biomolecular makeup, plays a crucial role in maintaining healthy cholangiocyte function and preserving the integrity of the biliary epithelium. Studies have demonstrated that the pH of bile and bicarbonate concentration during perfusion is able to predict the onset of nonanastomotic biliary strictures [51]. Consistently elevated bile pH (>7.45) and elevated bicarbonate concentrations (>18 mmol/L) decrease the risk of cholangiopathy posttransplant. Real-time sampling and close monitoring of bile production are major advantages of perfusion over SCS, providing a glimpse into both hepatocellular function and biliary tree health. This real-time risk stratification provides early awareness of grafts at risk for biliary complications before posttransplant morbidity/mortality are manifest, thus guiding organ utilization decisions.

### Novel biomarkers

MicroRNAs and distinct protein signatures in the perfusate are additional molecular markers being studied as potential graft function predictors [52]. MicroRNA-122 levels, for example, correlate strongly with hepatocellular injury; proteomics analysis also identified damage-associated molecular patterns that predict EAD. Perfusion has been used for metabolomic profile assessment [53], which allowed to visualize different profiles associated with organ quality or posttransplant outcomes. Some of these key metabolic signatures have been linked with metabolic disturbances leading to alterations in citric acid cycle intermediates and amino acid profiles which have predictive value for graft function posttransplant [12,45]. These newly developed biomarkers could serve as better-sensitive and -specific markers of graft viability [54]. High-throughput analysis of these molecular markers during perfusion could provide a more nuanced assessment of the organ and it may allow the development of predictive algorithms with multiple biomarker patterns.

### Integration of parameters

The assessment of viability has been refined by the integration of multiple parameters for the development of composite scoring systems [55]. Thus, machine learning algorithms trained on perfusion data also may serve to predict posttransplant outcomes [56]. Novel real-time assessment technologies that capture multiple parameters are emerging to facilitate clinical decision-making [57]. To that end, there are efforts internationally to try and standardize viability assessment criteria across perfusion platforms [58]. They would set uniform thresholds and assessment approaches that could be standardized across multiple centers and allow for evidence-based decisions on organ use.

## CLINICAL IMPLEMENTATION AND LOGISTICS OF MACHINE PERFUSION

Machine perfusion incorporation into clinical practice constitutes a dramatic shift in organ preservation requiring major changes to previously established transplant protocols and infrastructure. The transformation is multifaceted, covering not only technical prerequisites but also workforce development and economic aspects [59].

### Infrastructure and staffing needs for technical fund

Technical expertise and infrastructure requirements for machine perfusion demand dedicated facilities with advanced monitoring systems, controlled environments, and reliable backup systems [11]. Studies of leading transplant centers indicate optimal staffing requires 3–4 trained perfusionists providing round-the-clock coverage [60]. These specialists must combine perfusion expertise with comprehensive knowledge of liver physiology and transplant medicine [61]. Success depends on maintaining rigorous quality control, systematic data collection, and effective team coordination through standardized protocols and continuous training programs.

### Organ transport: logistical challenges

Machine perfusion transport poses distinct challenges beyond SCS, as demonstrated in a multicenter study of 250 cases [62]. Key requirements include specialized vehicles with mounting systems, reliable power supplies, environmental controls, and continuous monitoring capabilities. While transport protocols vary between centers [63], successful implementation depends on coordinated planning between procurement and recipient teams, with emphasis on temperature stability, backup systems, and portable perfusion devices [5].

### Economic implications and quality assurance

Machine perfusion requires substantial initial investment ($250 000–$500 000 per device) and ongoing costs ($10 000–15 000 per perfusion, plus training costs of $5000–8000 per team member) [64]; however, studies demonstrate potential cost-effectiveness through reduced hospital stays, 30% fewer complications, and 15% better 1-year graft survival rates [65], suggesting the improved clinical outcomes may offset the investment, though economic viability varies between healthcare systems. Successful machine perfusion programs rely on standardized protocols encompassing operating procedures, quality checkpoints, documentation requirements, and performance metrics [66]. While international consensus guidelines exist for standardization, regional variations persist [67], allowing centers to adapt protocols to their specific needs while maintaining core quality assurance standards and staff competencies.

### Training programs and competency assessment

Training programs incorporate theoretical education, simulation exercises, and supervised clinical experience [68], with competency assessment throughout. Studies show teams require 15–20 cases to achieve proficiency, with continued improvement through 50 cases [69]. The curriculum encompasses liver physiology, perfusion technology, and practical device operation, emphasizing ongoing education to maintain standards and keep pace with technological advances.

### Emergency response and risk management

Centers have developed comprehensive risk management strategies including backup systems, alternative preservation methods, and emergency protocols [21]. With equipment failure affecting 2–3% of cases [70], these measures encompass redundant critical components, routine maintenance, emergency drills, and strong relationships with manufacturers for technical support, ensuring continuous operation and rapid problem resolution.

### Conventional system integration

Machine perfusion implementation has necessitated revisions in organ allocation policies, sharing agreements, and documentation systems [71]. Changes include adjusted time parameters, enhanced transport logistics, and improved intercenter communication. The technology’s extended preservation capabilities enable better donor–recipient matching, while requiring comprehensive documentation of perfusion parameters and quality measures, leading to more specialized roles and standardized processes.

## THERAPEUTIC INTERVENTIONS DURING MACHINE PERFUSION

Machine perfusion has progressed from a simple preservation strategy to a platform for therapeutic applications. This represents a promising step toward organ optimization and rehabilitation pretransplantation [72].

### Pharmacological approaches

Machine perfusion is closely controlled, which allows for opportunities for localized pharmacological delivery and therapeutic interventions. Multiple studies have shown successful delivery of many different pharmacological agents during perfusion, as direct access to the organ vasculature allows for optimal drug distribution [73]. One group of promising therapies are anti-inflammatory treatments during perfusion, with studies demonstrating reduced ischemia-reperfusion injury and improved graft function [74]. Antioxidant therapy delivered during perfusion has been shown to exert considerable protection. During normothermic perfusion, the formulation of certain antioxidant therapies leads to decreased markers of oxidative stress [75], therefore improving posttransplant outcomes. Other studies on vasodilators and thrombolytic agents showed some changes in microvascular perfusion and vascular resistance improvement [76].

### Cell-based therapies

This novel combination of cell-based therapies and machine perfusion has emerged to improve organ rehabilitation. In experimental models, administration of mesenchymal stem cells during perfusion appears to provide benefit with improved renal graft function and reduced inflammatory responses [77]. Several studies have shown that these cells actively contribute to tissue repair processes at the time of the perfusion period [78]. Endothelial progenitor cells have been investigated in this regard and data have shown their capacity to restore vascular injury and enhance endothelial activity [79]. Special care is taken in assessing the timing and dosing of these cell-based therapies, with optimal administration protocols being observed during different phases of perfusion [80].

### Defatting protocols for steatotic livers

During normothermic perfusion, certain defatting protocols can effectively reduce hepatic fat content [81]. Such protocols use a combination of both metabolic modulators and pharmaceutical agents to promote lipid metabolism and export [82]. Targeted defatting therapies have shown significantly reduced liver fat content after only 6–12 h of perfusion [83]. These effects have been further elucidated in terms of their molecular mechanisms, with numerous studies demonstrating highly integrated cross-talk among different metabolic pathways [84]. Clinical trials conducted in recent years have shown improved outcomes using steatotic livers subjected to defatting protocols during perfusion [85]. The success of defatting interventions is temperature-sensitive, as treatments performed under normothermic conditions are more effective than those under hypothermic conditions [86]. Perioperatively monitoring defatting protocols has been shown by exploring specific biomarkers that can facilitate this process [87]. Defatting protocols combined with therapies such as hyperbaric oxygen, porcine gelatin sponge, and carnosine have produced synergistic effects and improved graft quality [88]. Novel strategies, such as incorporating gene therapy vectors during perfusion [89] and using targeted nanoparticle delivery systems, continue to be explored. There are evolving technologies that may offer more opportunities to enhance the therapeutic potential of machine perfusion within the field of liver transplantation [90].

## CONCLUSION: FUTURE PERSPECTIVES AND EMERGING TECHNOLOGIES IN MACHINE PERFUSION

Machine perfusion technology in liver transplantation has evolved significantly, transforming from a preservation method to a therapeutic platform [91]. Recent advances include automated perfusion control with real-time monitoring [92^■■^] and artificial intelligence-driven predictive modeling for organ viability assessment [93]. Technology enables therapeutic interventions such as defatting protocols for steatotic livers [94] and immunomodulation strategies [95], while stem cell therapies show promise for organ repair [96]. Advanced perfusion solutions incorporating oxygen carriers and protectants have demonstrated enhanced mitochondrial function [97] and targeted therapeutic additions offer new opportunities for graft improvement [98]. Extended preservation capabilities now exceed 24 h [99^■^], impacting organ-sharing policies [100], while innovative approaches combine perfusion with bioengineering techniques [101,102]. Ongoing standardization efforts [103,104] and focus on cost-effectiveness [105,106] are crucial for widespread implementation. Future developments point toward organ-specific protocols [107] and the integration of genomic and metabolomic data [108], suggesting a continued evolution of this transformative technology.

## Acknowledgements

We extend our deepest gratitude to the esteemed Italian-Americans, Leonardo Domiziano Zanza and Lucrezia Flavia Zanza, for their invaluable assistance in revising and editing our manuscript.

**Financial support and sponsorship**

*None.*

**Conflicts of interest**

*There are no conflicts of interest.*
